# Quantitative Flow Ratio Is Associated with Extent and Severity of Ischemia in Non-Culprit Lesions of Patients with Myocardial Infarction

**DOI:** 10.3390/jcm10194535

**Published:** 2021-09-30

**Authors:** Rosalia Dettori, Michael Frick, Kathrin Burgmaier, Richard Karl Lubberich, Martin Hellmich, Nikolaus Marx, Sebastian Reith, Mathias Burgmaier, Andrea Milzi

**Affiliations:** 1 Department of Cardiology, University Hospital of the RWTH Aachen, D-52070 Aachen, Germany; rdettori@ukaachen.de (R.D.); mfrick@ukaachen.de (M.F.); rlubberich@ukaachen.de (R.K.L.); nmarx@ukaachen.de (N.M.); sreith@ukaachen.de (S.R.); mburgmaier@ukaachen.de (M.B.); 2 Department of Pediatrics, University Hospital Cologne, D-50937 Cologne, Germany; kathrin.burgmaier@uk-koeln.de; 3 Institute of Medical Statistics and Computational Biology (IMSB), Faculty of Medicine and University Hospital Cologne, University of Cologne, D-50937 Cologne, Germany; martin.hellmich@uni-koeln.de

**Keywords:** coronary artery disease, quantitative flow ratio, cardiac magnetic resonance imaging, myocardial ischemia

## Abstract

Quantitative flow ratio (QFR) is a novel method to assess the relevance of coronary stenoses based only on angiographic projections. We could previously show that QFR is able to predict the hemodynamic relevance of non-culprit lesions in patients with myocardial infarction. However, it is still unclear whether QFR is also associated with the extent and severity of ischemia, which can effectively be assessed with imaging modalities such as cardiac magnetic resonance (CMR). Thus, our aim was to evaluate the associations of QFR with both extent and severity of ischemia. We retrospectively determined QFR in 182 non-culprit coronary lesions from 145 patients with previous myocardial infarction, and compared it with parameters assessing extent and severity of myocardial ischemia in staged CMR. Whereas ischemic burden in lesions with QFR > 0.80 was low (1.3 ± 5.5% in lesions with QFR ≥ 0.90; 1.8 ± 7.3% in lesions with QFR 0.81–0.89), there was a significant increase in ischemic burden in lesions with QFR ≤ 0.80 (16.6 ± 15.6%; *p* < 0.001 for QFR ≥ 0.90 vs. QFR ≤ 0.80). These data could be confirmed by other parameters assessing extent of ischemia. In addition, QFR was also associated with severity of ischemia, assessed by the relative signal intensity of ischemic areas. Finally, QFR predicts a clinically relevant ischemic burden ≥ 10% with good diagnostic accuracy (AUC 0.779, 95%-CI: 0.666–0.892, *p* < 0.001). QFR may be a feasible tool to identify not only the presence, but also extent and severity of myocardial ischemia in non-culprit lesions of patients with myocardial infarction.

## 1. Introduction

Quantitative flow ratio (QFR) is a novel method to assess hemodynamic relevance of coronary lesions based on a three-dimensional vessel reconstruction and estimation of its contrast media flow velocity [1,2,3,4]. In contrast to wire based methods such as fractional flow reserve (FFR) or instantaneous wave-free ratio (iFR), QFR does not require pressure wires or drug-induced hyperemia [1,2]. Several studies already demonstrated a good diagnostic performance of QFR in comparison to wire-based assessment of hemodynamic relevance of coronary lesions in the setting of chronic coronary syndromes [2,5,6,7,8]. Furthermore, we and others could demonstrate in large retrospective studies that QFR may be effectively used to evaluate the presence of ischemia in non-culprit lesions in the context of acute coronary syndromes [9,10,11,12]. QFR also shows a good concordance with stenosis geometry [13] and with non-invasive detection of ischemia, although the latter is less extensively explored [1,14,15].

Whereas the association of QFR with the sheer presence of myocardial ischemia has been consistently reported, it is still unclear whether this novel modality is also able to predict the extent of ischemia. This is of major clinical relevance since previous studies showed that the extent of ischemia correlates with patient outcome [16,17,18,19,20]. Furthermore, it is also unclear whether QFR can depict severity of ischemia, which may be assessed in CMR as relative intensity of ischemic areas, as previously described [21]. Therefore, this study aimed to assess the association of QFR of non-culprit lesions in patients with previous myocardial infarction with both extent and severity of myocardial ischemia, as assessed by CMR.

## 2. Materials and Methods

### 2.1. Study Population

We retrospectively enrolled 145 patients with previous myocardial infarction who underwent staged stress perfusion CMR within 1 year after intervention of the culprit lesion to determine hemodynamic relevance of the remaining coronary lesion(s) at the Department of Cardiology, University Hospital of the RWTH Aachen, Germany between the 1 January 2016 and the 1 June 2020. Previous studies partly included patients in this study cohort [12].

The main inclusion criterion was a previous myocardial infarction with one (or more) non treated, angiographically at least intermediate (with a lumen diameter stenosis ≥ 40%) non-culprit lesion, which was then further analyzed by stress-CMR to assess its hemodynamic relevance. Exclusion criteria were absence of CAD at baseline angiography, presence of 1-vessel CAD, absence of non-culprit lesions as defined above, relevant left main disease, previous CABG, cardiogenic shock during acute coronary angiography or direct indication to revascularization of the non-culprit vessel(s) by CABG or PCI without previous ischemia testing.

The study was approved by the local Ethics Committee and is in accordance with the Declaration of Helsinki on ethical principles for medical research involving human subjects.

### 2.2. QFR Analysis

A blinded certified investigator performed offline QFR using commercial software (QAngio XA 3D, Medis Medical Imaging System, Leiden, The Netherlands) according to a previously described protocol [5]. In short, QFR analysis required two angiographic projections at least 25° apart with minimal overlap. Retrospective data acquisition of coronary angiograms did not allow to apply an optimized image acquisition protocol. A minimal acquisition rate of 10 frames/second was necessary for inclusion. Further analysis was based on frame-counting QFR. 

### 2.3. CMR Image Acquisition and Analysis

CMR image acquisition was accomplished on a 1.5 Tesla magnetic resonance scanner (Achieva, Philips Healthcare, Best, The Netherlands). After standard cine imaging, contrast enhanced first pass perfusion imaging (3 short axis slices per heartbeat, intravenous bolus of Magnograf (Gadopentetat-Dimeglumin), 0.1 mmol/kg followed by 30 mL of saline flush at 4 mL/s) during vasodilator-stress with adenosine (140 µg/kg/min intravenously for 4 min) took place. Standard late gadolinium enhancement (LGE) imaging was performed 10 min after contrast injection (a second portion was given to add up to 0.2 mmol/kg).

An experienced, board-certified CMR cardiologist, blinded to the results of the QFR examination, performed CMR image analysis on a dedicated CMR workstation (ExtendedWorkspace, Philips Healthcare, Best, The Netherlands), as previously described [21].

The dynamic of the first pass perfusion sequence with the greatest extent of ischemia was chosen and the apical, mid-ventricular and basal short axis (SA) slice of this dynamic were established for further analysis. Single analyses were strictly separated when several ischemic areas attributable to different coronary artery supply territories were detected. 

For scar quantification, a corresponding SA-slice of late gadolinium enhancement (LGE)-imaging for each slice of perfusion was defined. 

**Figure 1:**
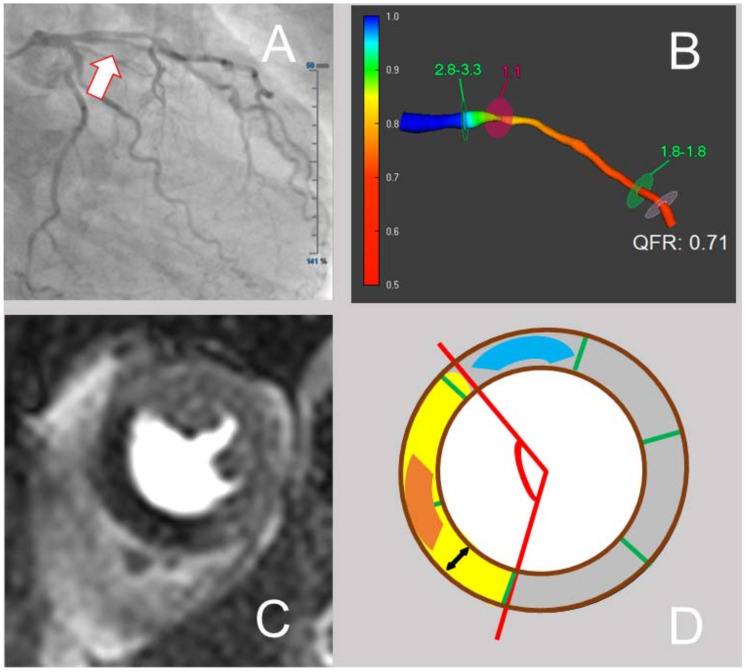
Exemplificative analysis of angiography, QFR and CMR images. In (**A**), angiographic image of a STEMI patient with occlusion of RCA (not shown) and non-culprit, intermediate stenosis of the LAD (marked with a white arrow) is shown. In (**B**), QFR analysis of the hemodynamic relevance of the non-culprit LAD stenosis is shown. In (**C**), a contrast enhanced first pass perfusion imaging of the mid-ventricular layer with myocardial ischemia of the anteroseptal/septal segments is presented. In (**D**), schematized analysis of this CMR image is shown. Here, myocardial area is delimited by the brown border, ischemic area is shown in yellow; signal intensity of ischemia was measured in the center (orange) of the ischemic area, signal intensity of remote myocardium was measured in the non-ischemic area (blue); circumferential extent of ischemia (red) corresponded to the angle of ischemia with vertex at the center of the AHA 16 segment model.

For each SA-slice, myocardial area was calculated by manually tracking the endocardial and the epicardial borders. Classification of the myocardium of each slice relied on the standardized AHA 16 segment model [22]. For each slice, visual assessment of the region of ischemia was performed by excluding an area of scar within an ischemic region. The percentage of ischemic burden was calculated as ischemic area divided by the total myocardial area × 100. Circumferential extent of ischemia was measured by defining the center of the 16-segment AHA-model as the vertex of the angle of ischemia and then manually tracing the ischemic angle. Intensity of ischemia was quantified by calculating the “intensity of myocardial ischemia index”. For this purpose, the signal intensity of the darkest area (excluding a probable dark rim artifact at the endocardial border) within the ischemic area, as well as the signal intensity of a remote myocardial area (as visually assessed) were determined. The intensity of myocardial ischemia index corresponded to [1-(signal intensity ischemia/signal intensity remote)] × 100. An exemplificative analysis of CMR images is shown in Figure 1.

### 2.4. Statistical Analysis

Categorical variables were summarized as count (percentage), continuous variables as mean ± standard deviation. Analysis of variance (ANOVA) was used to analyze the distribution of various parameters assessing extent and severity of ischemia among predefined groups of QFR-values (≥0.90; 0.81–0.89; ≤0.80). In order to test the distribution of extent and severity of ischemia expressed as ordinal variables throughout these predefined groups of QFR-values, we employed Kruskal–Wallis H-test; the results of the following post hoc analyses are reported after Bonferroni correction for multiple testing.

Receiver operating curve (ROC) analysis was performed to identify the diagnostic efficiency of QFR in predicting a clinically relevant myocardial ischemia, defined as an ischemic burden ≥10% of viable myocardium, as previously described [19,23]. The diagnostic efficiency according to the values of the area under the curve (AUC) was classified as described elsewhere [24]. QFR value with the highest Youden index (sensitivity + specificity − 1) was defined as optimal cut-off-value for prediction of clinically relevant ischemia.

All statistical analyses were performed with SPSS software v 26.0 (IBM Corp., Armonk, NY, USA). A *p*-value < 0.05 indicated statistical significance.

## 3. Results

### 3.1. Clinical Characteristics

We retrospectively analyzed a total of 182 non-culprit lesions of at least intermediate severity (mean percent diameter stenosis: 46 ± 9%) from 145 patients with preceding myocardial infarction who underwent staged stress perfusion CMR to assess hemodynamic relevance of the remaining non-culprit lesion(s). For patient and lesion characteristics please refer to Table 1 and Table 2. Accuracy of QFR ≤ 0.80 in predicting the presence of ischemia in staged CMR was 87.9%.

### 3.2. QFR and CMR Parameters in the Assessment of Both Extent and Severity of Myocardial Ischemia

**Figure 2:**
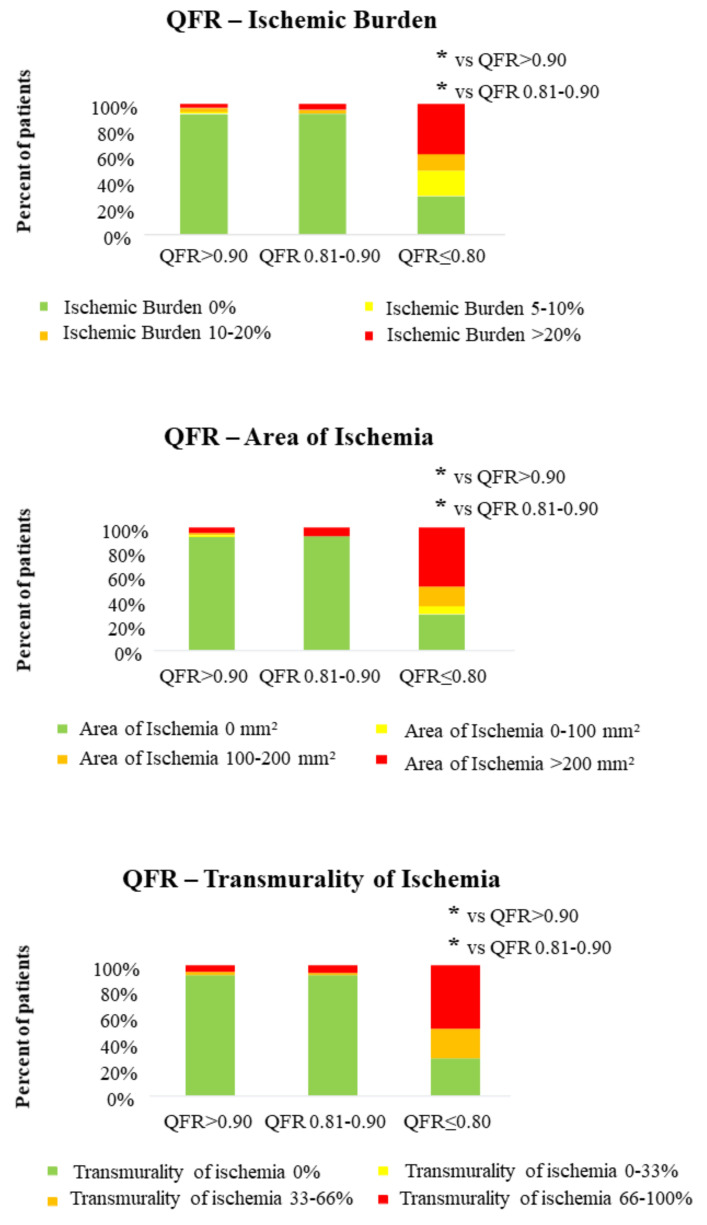
QFR is associated with extent of myocardial ischemia in CMR. Throughout predefined groups of QFR, the distribution of parameters assessing extent of ischemia was significantly different (*p* < 0.001 for all three parameters); relevant differences in the pairwise comparison are reported with an asterisk (* for *p* < 0.001).

First, we aimed to study whether QFR is associated with CMR parameters assessing extent of ischemia in our study population. Whereas ischemic burden in both groups with QFR > 0.80 was low (1.3 ± 5.5% in lesions with QFR ≥ 0.90; 1.8 ± 7.3% in lesions with QFR 0.81–0.89), there was a significant increase in ischemic burden in lesions with QFR ≤ 0.80 (16.6 ± 15.6%; *p* < 0.001 for distribution, *p* < 0.001 for group 1 vs. group 3). These data were confirmed by all other parameters defining extent of ischemia (ischemic area, transmurality of ischemia, circumferential extent of ischemia), as shown in Table 3. A graphical representation of this distribution is presented in Figure 2. Furthermore, among a small group of patients (*n* = 8) with very low QFR lesions (QFR ≤ 0.70), 50% demonstrated extensive ischemia with ischemic burden > 20% and 62.5% even presented an ischemia with a transmurality > 66%.

**Figure 3:**
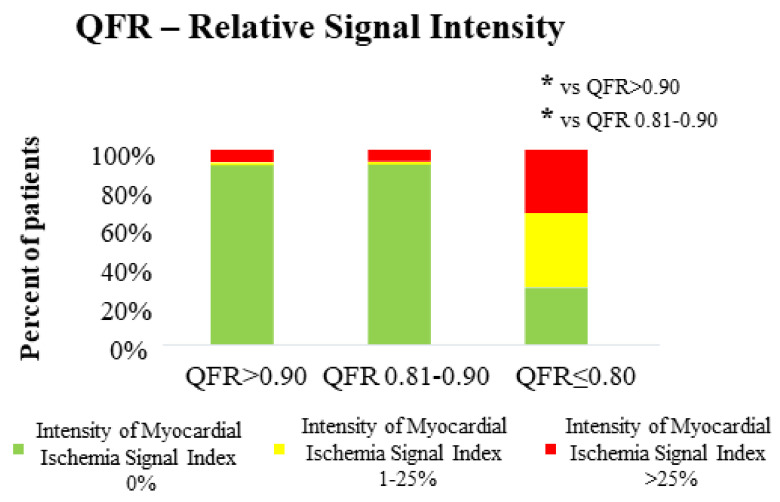
Graphic illustration of the association of intensity of myocardial ischemia index and QFR value of the respective non-culprit lesion. A bar graph illustrates the correlation between relative intensity of myocardial ischemia and QFR-groups (* for *p* < 0.001).

After showing a significant association of QFR with extent of ischemia, we tested whether QFR is also associated with ischemia severity, as expressed by relative intensity of ischemic areas. CMR-derived intensity of myocardial ischemia index was associated with QFR of the respective non-culprit lesion (3.0 ± 12.0% in lesions with QFR ≥ 0.90; 2.0 ± 9.3% in lesions with QFR 0.81–0.89; 22.0 ± 20.1% in lesions with QFR ≤0.80; *p* < 0.001 for distribution, *p* < 0.001 for comparison group 1 vs. group 3) (Table 3). The distribution is also graphically depicted in Figure 3. Similar to the results concerning extent of ischemia, the group with very low QFR values (QFR ≤ 0.70) presented a numerically higher percentage of very severe ischemia (32.3%), defined as an intensity of myocardial ischemia index > 25%.

### 3.3. Diagnostic Efficiency of QFR in Predicting Clinically Relevant Ischemia ≥ 10%

**Figure 4:**
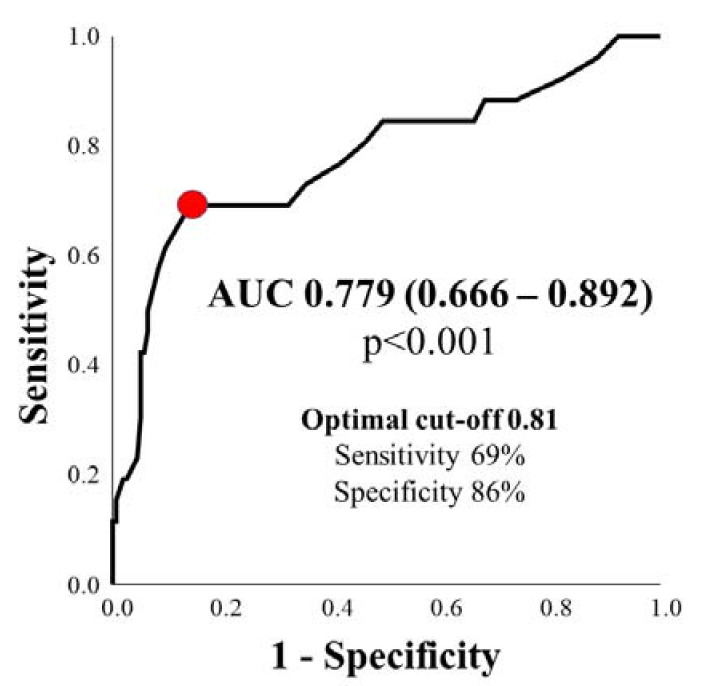
QFR predicts myocardial ischemia ≥10% with good diagnostic efficiency. AUC = area under the curve.

After demonstrating a robust association of QFR with CMR-derived parameters assessing extent and severity of ischemia, we aimed to test the diagnostic efficiency of QFR for the detection of a clinically relevant ischemia, defined as an ischemic burden ≥10% [19,23]. QFR could predict myocardial ischemia ≥10% of non-culprit lesions with a good accuracy (AUC 0.779, 95%-CI: 0.666–0.892, *p* < 0.001). Optimal cut-off for QFR in the prediction of myocardial ischemia ≥10% was 0.81 (sensitivity = 69%, specificity = 86%), as shown in Figure 4.

## 4. Discussion

The main finding of our study is that QFR is associated with both extent and severity of myocardial ischemia in non-culprit lesions of patients with myocardial infarction.

QFR represents a reliable, non-invasive tool in the assessment of hemodynamic relevance of coronary lesions not only in the setting of chronic, but also acute coronary syndromes [11,25,26,27,28]. So far, only a limited number of studies compared the diagnostic accuracy of QFR with non-invasive myocardial perfusion imaging modalities, achieving in part discordant results. Lenk et al. recently revealed a high association between QFR and myocardial ischemia assessed by stress CMR with a diagnostic accuracy of 86% [14]. In line with these observations, Smit et al. showed an overall accuracy of 90% of QFR in the detection of myocardial ischemia on single-photon emission computed tomography myocardial perfusion imaging (SPECT) [15]. Both these studies included a population with chronic coronary syndromes and a similar risk profile. In contrast, Sejr-Hansen et al. described only a modest concordance of FFR/QFR with SPECT or CMR in patients with suspected coronary artery disease in coronary computed tomography angiography [1]. However, this difference may be due to different patient inclusion criteria and due to a higher proportion of lesions in the QFR “grey zone” compared with the previous studies. In a previous analysis, our group could show that QFR based on acute angiograms is able to assess the presence of ischemia in non-culprit lesions of patients with myocardial infarction [12]. All these previous studies, though, only assessed results of myocardial perfusion imaging in terms of presence/absence of myocardial ischemia. Furthermore, it is still unknown whether QFR is also able to quantitatively assess extent and/or severity of ischemic myocardium; this is especially important as an ischemic area ≥10% of myocardium is associated with a worse prognosis [16,17,19,20,29]. Our study now extends current knowledge by showing that a lower QFR in non-culprit lesions of patients with previous myocardial infarction is associated with larger areas of ischemic myocardium. Similar data could be previously reported for FFR, although in a smaller population with chronic coronary syndrome and advanced coronary stenoses [30]. Lower QFR values were also associated with a more pronounced ischemia, as shown by the higher relative intensity of myocardial ischemia index of ischemic areas in CMR.

Our data represent a further, independent validation of QFR as a method to quantitatively assess coronary flow. In fact, one of the main determinants of a larger ischemic area is represented by the magnitude of flow reduction determined by a coronary stenosis; this more pronounced flow reduction is characterized by lower QFR values. 

In spite of the good association of QFR with extent and severity of ischemia in CMR, it has to be noticed that a complete overlap cannot be expected. In fact, QFR does not consider some determinants of myocardial ischemia, such as coronary artery dominance, presence of collaterals or ischemic preconditioning, which may partly explain some diverging results. In particular, in our study a relevant proportion (29%) of patients with a pathologic QFR does not show any relevant ischemia in CMR; this may be due to the factors mentioned above and may also represent a limitation of QFR in the setting of acute myocardial infarction. Furthermore, as previously reported, a diffuse coronary artery disease, which may bear a worse prognosis, can result in a comparatively small extent of ischemia visible in CMR (as ischemia is balanced) in comparison with a single high-grade coronary stenosis with a larger area of ischemia [20].

As QFR allows a good estimation of extent and severity of ischemia may prompt clinicians to use this tool in order to select lesions with a more urgent need for treatment. In fact, the presence of large ischemic areas is a predictor of adverse outcome [16,17,18,19,20]. Recognizing these lesions and prioritizing their treatment may allow a better resource allocation and improve patient outcome. Notably, clinicians would be able to gather such relevant information in an inexpensive fashion by simply analyzing acute coronary angiographies. However, it has to be noticed that our study only included non-culprit lesions which, based on clinical judgement, were selected for further ischemia testing based on their possible prognostic relevance and therefore may be biased; this implies that the reported associations are valid only for proximal or medial stenoses of major vessels, and that our conclusion cannot be extended to small or peripheral vessels, which obviously supply much smaller areas and may have a much lower clinical impact. Furthermore, our study is unable to assess the impact of our findings on prognosis, and overall the role of flow evaluation in the guidance of revascularization in non-culprit lesions of patients with infarction has been recently questioned following the publication of the results of the FLOWER-MI study [31]. Here, no relevant benefit of a FFR-guided complete revascularization over angiography could be detected. Further ongoing studies, such as the FIRE trial [32], are warranted to answer this question, assessing also the role of QFR in the evaluation of non-culprit lesions in the context of myocardial infarction.

Although being, to the best of our knowledge, the first study assessing the association of QFR with both extent and severity of ischemia, some limitations have to be reported. First, due to the study design we only included non-culprit lesions of patients with myocardial infarction; although previous data suggest a similar accuracy of QFR in both chronic and acute coronary syndromes, we are unable to generalize our conclusions to the population with chronic coronary syndromes. Although showing a good association of QFR with extent and severity of ischemia, we cannot exclude a potential selection bias, due to the inclusion of the lesions which were deemed relevant at the interventionalists’ discretion, which naturally exclude very peripheral or very small vessels. Moreover, our data need to be confirmed in larger prospective cohorts.

## 5. Conclusions

QFR is associated not only to the presence, but also the extent and severity of myocardial ischemia in non-culprit lesions of patients with myocardial infarction.

## Figures and Tables

**Table 1:** Patient characteristics. Abbreviations: BMI = body mass index; LDLc = low density lipoprotein cholesterol; HDLc = high density lipoprotein cholesterol; LAD = left anterior descending; LCx = left circumflex; RCA = right coronary artery; RIM = ramus intermedius.

	*n* = 145
Age (years)	64.2 ± 15.0
Male sex (*n*, %)	114 (78.6)
STEMI at initial presentation (*n*, %)	58 (40)
NSTEMI at initial presentation (*n*,%)	87 (60)
Detection of ischemia (*n*, %)	26 (17.9)
LV-EF (%)	49.9 ± 8.0
**CV Risk profile**	
Diabetes mellitus (*n*, %)	38 (27.0)
BMI (kg/m^2^)	26.9 ± 3.5
Hypertension (*n*, %)	86 (61.4)
Current smoking (*n*, %)	49 (36.0)
Pack Years (PY)	27.3 ± 24.5
**Lab values**	
Cholesterol (mg/dL)	212.2 ± 147.0
LDLc (mg/dL)	140.9 ± 47.6
HDLc (mg/dL)	46.6 ± 13.5
Triglycerides (mg/dL)	109.9 ± 95.2
HbA1c (%)	6.1 ± 1.3
**Non-culprit lesion (vessel)**	
LAD (*n*, %)	67 (46.2)
LCx (*n*, %)	35 (24.1)
RCA (*n*, %)	33 (22.8)
Diagonal branch (*n*, %)	4 (2.8)
Obtuse branch (*n*, %)	5 (3.4)
RIM (*n*, %)	1 (0.7)

**Table 2:** QFR-derived lesion characteristics. Abbreviations: MLD = minimal lumen diameter.

	*n* = 182
**QFR-derived stenosis parameters**	
QFR	0.86 ± 0.08
MLD (mm)	1.36 ± 0.39
Percent area stenosis (%)	45.4 ± 9.0
Lesion length (mm)	23.9 ± 13.8

**Table 3:** Extent and severity of ischemia in QFR-groups.

	Group 1	Group 2	Group 3	*p*	*p*_Group3vs.1_
	QFR ≥ 0.90	QFR 0.81–0.90	QFR ≤ 0.80		
Area of ischemia (mm^2^)	21.8 ± 85.1	26.3 ± 101.8	265.6 ± 249.0	<0.001	<0.001
Ischemic burden (%)	1.3 ± 5.5	1.8 ± 7.3	16.6 ± 15.6	<0.001	<0.001
Transmurality of ischemia (%)	5.6 ± 20.2	5.7 ± 21.1	53.5 ± 37.2	<0.001	<0.001
Circumferential extent of ischemia (°)	3.4 ± 12.3	5.8 ± 22.3	47.3 ± 35.2	<0.001	<0.001
Intensity of myocardial ischemia index (%)	3.0 ± 12.0	2.0 ± 9.3	22.0 ± 20.1	<0.001	<0.001

## Data Availability

Data are available on reasonable request to the corresponding author.
